# Supplementary material for: A Computational Approach to Evaluate the Combined Effect of SARS-CoV-2 RBD Mutations and ACE2 Receptor Genetic Variants on Infectivity: The COVID-19 Host-Pathogen Nexus
Source: Front Cell Infect Microbiol. 2021 Aug 9;11:707194. doi: 10.3389/fcimb.2021.707194 (PMC8381355; doi:10.3389/fcimb.2021.707194)
Supplement: Supplementary file 5 [file Table_1.pdf]

Supplementary Table 1: Changes in the polar (red P) and hydrophobic (green H) interactions between spike RBD and hACE2 receptor contact resides in different mutated strains of SARS-CoV-2. Note: only missing, changed and new interactions are mentioned under each mutation, all other interactions are same as the Wuhan strain.10 out of 18 contact residues of spike RBD show polar interactions with hACE2 receptor (highlighted in red). (*)= salt bridge

**Spike RBD Wuhan strain contact residues**	**hACE2 contact residues**	**Mutants**												
		K417N	K417R	G446A	Y449N	Y453F	L455F	F456L	A475V	G476S	G476A	F486L	Q493R	Q493L
Lys417	1X Asp30(P)<br>1X Asp30(H)	Missing<br>Missing	4X Asp30(H)											
Gly446	1X Gln42(P)			Missing										
Tyr449	1X Asp38(P)<br>1X Gln42(P)<br>3X Asp38(H)				Missing<br>Missing<br>Missing									
Tyr453	1X His34(P)<br>2X His34(H)					Missing<br>Missing								
Leu455	4X His34(H)					1X His34(H)	4X Asp30(H)<br>1X Lys31(H)							
Phe456	1X Thr27(H)<br>1X Asp30(H)							Missing						
Ala475	1X Ser19(P)<br>2X Ser19(H)<br>1X Gln24(H)								2X Gln24(H)<br>2X Thr27(H)					
Gly476	1X Ser19(H)									Missing	Missing			
Phe486	1X Met82(H)<br>4X Tyr83(H)											1X Tyr83(H)		
Asn487	1X Gln24(P)<br>1X Tyr83(P)<br>6X Gln24(H)<br>3X Tyr83(H)													
Tyr489	1X Thr27(H)<br>1X Phe28(H)													
Gln493	2X His34(H)<br>1X Glu35(H)					1X His34(H)							1X Glu35 (P)<br>Missing<br>4X Glu35(H)	1X His34(H)<br>Missing<br>1X Lys31(H)
Gly496	1X Lys353(P)<br>1X Asp38(H)<br>2X Lys353(H)													
Gln498	1X Gln42(P)<br>3X Tyr41(H)<br>2X Gln42(H)<br>1X Leu45(H)													
Thr500	1X Tyr41(P)<br>3X Tyr41(H)<br>1X Asn330(H)<br>2X Asp355(H)<br>2X Arg357(H)													
Asn501	3X Tyr41(H)<br>1X Lys353(H)													
Gly502	1X Lys353(P)<br>1X Lys353(H)<br>2X Gly354(H)													
Tyr505	5X Lys353(H)<br>1X Gly354(H)													

Supplementary Table 1: (Continued)

Spike RBD Wuhan strain contact residues	hACE2 contact residues	Mutants									
		G496C	T500I	N501T	N501S	N501Y	G502D	G502C	G502R	Y505H	Y505E
Lys417	1X Asp30(P)<br>1X Asp30(H)										
Gly446	1X Gln42(P)										
Tyr449	1X Asp38(P)<br>1X Gln42(P)<br>3X Asp38(H)										
Tyr453	1X His34(P)<br>2X His34(H)										
Leu455	4X His34(H)										
Phe456	1X Thr27(H)<br>1X Asp30(H)										
Ala475	1X Ser19(P)<br>2X Ser19(H)<br>1X Gln24(H)										
Gly476	1X Ser19(H)										
Phe486	1X Met82(H)<br>4X Tyr83(H)										
Asn487	1X Gln24(P)<br>1X Tyr83(P)<br>6X Gln24(H)<br>3X Tyr83(H)										
Tyr489	1X Thr27(H)<br>1X Phe28(H)										
Gln493	2X His34(H)<br>1X Glu35(H)										
Gly496	1X Lys353(P)<br>1X Asp38(H)<br>2X Lys353(H)	7X Asp38(H)									
Gln498	1X Gln42(P)<br>3X Tyr41(H)<br>2X Gln42(H)<br>1X Leu45(H)		2X Tyr41(H)								
Thr500	1X Tyr41(P)<br>3X Tyr41(H)<br>1X Asn330(H)<br>2X Asp355(H)<br>2X Arg357(H)		Missing<br>1X Tyr41(H)<br><br>1X Asp355(H)<br>1X Arg357(H)<br>1X Leu45(H)								
Asn501	3X Tyr41(H)<br>1X Lys353(H)			2X Tyr41(H)<br>3X Lys353(H)	1X Tyr41(H)<br>3X Lys353(H)	5X Tyr41(H)<br>6X Lys353(H)					
Gly502	1X Lys353(P)<br>1X Lys353(H)<br>2X Gly354(H)						2X Lys353(H)<br>4X Gly354(H)<br>1X Thr324(H)	2X Lys353(H)<br>4X Gly354(H)	4X Gly354(H)<br>3X Thr324(H)		
Tyr505	5X Lys353(H)<br>1X Gly354(H)									2X Lys353(H)<br>Missing	7X Lys353(H)<br>Missing<br>***1X Lys353 (*)***
