# Supplementary material for: A Computational Approach to Evaluate the Combined Effect of SARS-CoV-2 RBD Mutations and ACE2 Receptor Genetic Variants on Infectivity: The COVID-19 Host-Pathogen Nexus
Source: Front Cell Infect Microbiol. 2021 Aug 9;11:707194. doi: 10.3389/fcimb.2021.707194 (PMC8381355; doi:10.3389/fcimb.2021.707194)
Supplement: Supplementary file 6 [file Table_2.pdf]

Supplementary Table 2: Changes in the polar and hydrophobic interactions in 24 different SARS-CoV-2 spike RBD mutations that occurred in the 18 contact residues with nine hACE2 genetic variants. P: polar, H: hydrophobic. Only missing, changed and new interactions are mentioned for each variant. (*)= salt bridge.

Spike RBD Wuhan strain contact residues	hACE2 isoform 1 contact residues	Wuhan Strain							
		D355A	D355N	E35D	E35K	F40L	M82I	T27A	S19P
Lys417	1X Asp30(P)<br>1X Asp30(H)								
Gly446	1X Gln42(P)								
Tyr449	1X Asp38(P)<br>1X Gln42(P)<br>3X Asp38(H)								
Tyr453	1X His34(P)<br>2X His34(H)								
Leu455	4X His34(H)								
Phe456	1X Thr27(H)<br>1X Asp30(H)							1X Ala27(H)	
Ala475	1X Ser19(P)<br>2X Ser19(H)<br>1X Gln24(H)								Missing<br>1XPro19(H)
Gly476	1X Ser19(H)								1XPro19(H)
Phe486	1X Met82(H)<br>4X Tyr83(H)						1X Ile82(H)		
Asn487	1X Gln24(P)<br>1X Tyr83(P)<br>6X Gln24(H)<br>3X Tyr83(H)								
Tyr489	1X Thr27(H)<br>1X Phe28(H)							Messing	
Gln493	2X His34(H)<br>1X Glu35(H)			1XAsp35(P)<br>1X Asp35(H)	1X Lys35(H)				
Gly496	1X Lys353(P)<br>1X Asp38(H)<br>2X Lys353(H)								
Gln498	1X Gln42(P)<br>3X Tyr41(H)<br>2X Gln42(H)<br>1X Leu45(H)								
Thr500	X Tyr41(P)<br>3X Tyr41(H)<br>1X Asn330(H)<br>2X Asp355(H)<br>2X Arg357(H)	1X Ala355(H)	1X Asn355(P)<br>4x Asn355(H)						
Asn501	3X Tyr41(H)<br>1X Lys353(H)		1X Asn355(H)						
Gly502	1X Lys353(P)<br>1X Lys353(H)<br>2X Gly354(H)								
Tyr505	5X Lys353(H)<br>1X Gly354(H)								

Supplementary Table 2: (Continued)

Spike RBD Wuhan strain contact residues	hACE2 isoform 1 contact residues	K417N							
		D355A	D355N	E35D	E35K	F40L	M82I	T27A	S19P
Lys417	1X Asp30(P)<br>1X Asp30(H)	Missing<br>Missing	Missing<br>Missing	Missing<br>Missing	Missing<br>Missing	Missing<br>Missing	Missing<br>Missing	Missing<br>Missing	Missing<br>Missing
Gly446	1X Gln42(P)								
Tyr449	1X Asp38(P)<br>1X Gln42(P)<br>3X Asp38(H)								
Tyr453	1X His34(P)<br>2X His34(H)								
Leu455	4X His34(H)								
Phe456	1X Thr27(H)<br>1X Asp30(H)								
Ala475	1X Ser19(P)<br>2X Ser19(H)<br>1X Gln24(H)								Missing<br>1XPro19(H)
Gly476	1X Ser19(H)								
Phe486	1X Met82(H)<br>4X Tyr83(H)						1X Ile82(H)		
Asn487	1X Gln24(P)<br>1X Tyr83(P)<br>6X Gln24(H)<br>3X Tyr83(H)								
Tyr489	1X Thr27(H)<br>1X Phe28(H)							Missing	
Gln493	2X His34(H)<br>1X Glu35(H)			1X Asp35(P)<br>1X Asp35(H)	1X Lys35(H)				
Gly496	1X Lys353(P)<br>1X Asp38(H)<br>2X Lys353(H)								
Gln498	1X Gln42(P)<br>3X Tyr41(H)<br>2X Gln42(H)<br>1X Leu45(H)								
Thr500	1X Tyr41(P)<br>3X Tyr41(H)<br>1X Asn330(H)<br>2X Asp355(H)<br>2X Arg357(H)	1X Ala355(H)	1X Asn355(P)<br>4X Ala355(H)						
Asn501	3X Tyr41(H)<br>1X Lys353(H)		1X Ala355(H)						
Gly502	1X Lys353(P)<br>1X Lys353(H)<br>2X Gly354(H)								
Tyr505	5X Lys353(H)<br>1X Gly354(H)								

Supplementary Table 2: (Continued)

Spike RBD Wuhan strain contact residues	hACE2 isoform 1 contact residues	K417R							
		D355A	D355N	E35D	E35K	F40L	M82I	T27A	S19P
Lys417	1X Asp30(P)<br>1X Asp30(H)	4X Asp30(H)	4X Asp30(H)	4X Asp30(H)	4X Asp30(H)	4X Asp30(H)	4X Asp30(H)	4X Asp30(H)	4X Asp30(H)
Gly446	1X Gln42(P)								
Tyr449	1X Asp38(P)<br>1X Gln42(P)<br>3X Asp38(H)								
Tyr453	1X His34(P)<br>2X His34(H)								
Leu455	4X His34(H)								
Phe456	1X Thr27(H)<br>1X Asp30(H)							1X Ala7(H)	
Ala475	1X Ser19(P)<br>2X Ser19(H)<br>1X Gln24(H)								Missing<br>1X Pro19(H)
Gly476	1X Ser19(H)								1X Pro19(H)
Phe486	1X Met82(H)<br>4X Tyr83(H)						1X Ile82(H)		
Asn487	1X Gln24(P)<br>1X Tyr83(P)<br>6X Gln24(H)<br>3X Tyr83(H)								
Tyr489	1X Thr27(H)<br>1X Phe28(H)							Missing	
Gln493	2X His34(H)<br>1X Glu35(H)			1X Asp35(P)<br>1X Asp35(H)	1X Lys35(H)				
Gly496	1X Lys353(P)<br>1X Asp38(H)<br>2X Lys353(H)								
Gln498	1X Gln42(P)<br>3X Tyr41(H)<br>2X Gln42(H)<br>1X Leu45(H)								
Thr500	1X Tyr41(P)<br>3X Tyr41(H)<br>1X Asn330(H)<br>2X Asp355(H)<br>2X Arg357(H)	1X Ala355(H)	1X Asn355(P)<br>4X Asn355(H)						
Asn501	3X Tyr41(H)<br>1X Lys353(H)		1X Asn355(H)						
Gly502	1X Lys353(P)<br>1X Lys353(H)<br>2X Gly354(H)								
Tyr505	5X Lys353(H)<br>1X Gly354(H)								

Supplementary Table 2: (Continued)

Spike RBD Wuhan strain contact residues	hACE2 isoform 1 contact residues	G446A							
		D355A	D355N	E35D	E35K	F40L	M82I	T27A	S19P
Lys417	1X Asp30(P)<br>1X Asp30(H)								
Gly446	1X Gln42(P)	Missing	Missing	Missing	Missing	Missing	Missing	Missing	Missing
Tyr449	1X Asp38(P)<br>1X Gln42(P)<br>3X Asp38(H)								
Tyr453	1X His34(P)<br>2X His34(H)								
Leu455	4X His34(H)								
Phe456	1X Thr27(H)<br>1X Asp30(H)							1X Ala27(H)	
Ala475	1X Ser19(P)<br>2X Ser19(H)<br>1X Gln24(H)								Missing<br>1X Pro19(H)
Gly476	1X Ser19(H)								1X Pro19(H)
Phe486	1X Met82(H)<br>4X Tyr83(H)						1X Ile82(H)		
Asn487	1X Gln24(P)<br>1X Tyr83(P)<br>6X Gln24(H)<br>3X Tyr83(H)								
Tyr489	1X Thr27(H)<br>1X Phe28(H)							Missing	
Gln493	2X His34(H)<br>1X Glu35(H)			1X Asp35 (P)<br>1X Asp35(H)	1X Lys35(H)				
Gly496	1X Lys353(P)<br>1X Asp38(H)<br>2X Lys353(H)								
Gln498	1X Gln42(P)<br>3X Tyr41(H)<br>2X Gln42(H)<br>1X Leu45(H)								
Thr500	1X Tyr41(P)<br>3X Tyr41(H)<br>1X Asn330(H)<br>2X Asp355(H)<br>2X Arg357(H)	1X Aal355(H)	1X Asn355(P)<br>4X Aal355(H)						
Asn501	3X Tyr41(H)<br>1X Lys353(H)		1X Aal355(H)						
Gly502	1X Lys353(P)<br>1X Lys353(H)<br>2X Gly354(H)								
Tyr505	5X Lys353(H)<br>1X Gly354(H)								

Supplementary Table 2: (Continued)

Spike RBD Wuhan strain contact residues	hACE2 isoform 1 contact residues	Y449N							
		D355A	D355N	E35D	E35K	F40L	M82I	T27A	S19P
Lys417	1X Asp30(P)<br>1X Asp30(H)								
Gly446	1X Gln42(P)								
Tyr449	1X Asp38(P)<br>1X Gln42(P)<br>3X Asp38(H)	Missing<br>Missing<br>Missing	Missing<br>Missing<br>Missing	Missing<br>Missing<br>Missing	Missing<br>Missing<br>Missing	Missing<br>Missing<br>Missing	Missing<br>Missing<br>Missing	Missing<br>Missing<br>Missing	Missing<br>Missing<br>Missing
Tyr453	1X His34(P)<br>2X His34(H)								
Leu455	4X His34(H)								
Phe456	1X Thr27(H)<br>1X Asp30(H)							1X Ala27(H)	
Ala475	1X Ser19(P)<br>2X Ser19(H)<br>1X Gln24(H)								Missing<br>1X Pro19(H)
Gly476	1X Ser19(H)								1X Pro19(H)
Phe486	1X Met82(H)<br>4X Tyr83(H)						1X Ile82(H)		
Asn487	1X Gln24(P)<br>1X Tyr83(P)<br>6X Gln24(H)<br>3X Tyr83(H)								
Tyr489	1X Thr27(H)<br>1X Phe28(H)							Missing	
Gln493	2X His34(H)<br>1X Glu35(H)			1X Asp35 (P)<br>1X Asp35(H)	1X Lys35(H)				
Gly496	1X Lys353(P)<br>1X Asp38(H)<br>2X Lys353(H)								
Gln498	1X Gln42(P)<br>3X Tyr41(H)<br>2X Gln42(H)<br>1X Leu45(H)								
Thr500	1X Tyr41(P)<br>3X Tyr41(H)<br>1X Asn330(H)<br>2X Asp355(H)<br>2X Arg357(H)	1X Ala355(H)	1X Asn355 (P)<br>4X Asn355(H)						
Asn501	3X Tyr41(H)<br>1X Lys353(H)		1X Asn355(H)						
Gly502	1X Lys353(P)<br>1X Lys353(H)<br>2X Gly354(H)								
Tyr505	5X Lys353(H)<br>1X Gly354(H)								

Supplementary Table 2: (Continued)

Spike RBD Wuhan strain contact residues	hACE2 isoform 1 contact residues	Y453F							
		D355A	D355N	E35D	E35K	F40L	M82I	T27A	S19P
Lys417	1X Asp30(P)<br>1X Asp30(H)								
Gly446	1X Gln42(P)								
Tyr449	1X Asp38(P)<br>1X Gln42(P)<br>3X Asp38(H)								
Tyr453	1X His34(P)<br>2X His34(H)	Missing<br>Missing	Missing<br>Missing	Missing<br>Missing	Missing<br>Missing	Missing<br>Missing	Missing<br>Missing	Missing<br>Missing	Missing<br>Missing
Leu455	4X His34(H)	1X His34(H)	1X His34(H)	1X His34(H)	1X His34(H)	1X His34(H)	1X His34(H)	1X His34(H)	1X His34(H)
Phe456	1X Thr27(H)<br>1X Asp30(H)							1X Ala27(H)	
Ala475	1X Ser19(P)<br>2X Ser19(H)<br>1X Gln24(H)								Missing<br>1X Pro19(H)
Gly476	1X Ser19(H)								1X Pro19(H)
Phe486	1X Met82(H)<br>4X Tyr83(H)						1X Ile82(H)		
Asn487	1X Gln24(P)<br>1X Tyr83(P)<br>6X Gln24(H)<br>3X Tyr83(H)								
Tyr489	1X Thr27(H)<br>1X Phe28(H)							Missing	
Gln493	2X His34(H)<br>1X Glu35(H)	1X His34(H)	1X His34(H)	1X Asp35(P)<br>1X His34(H)<br>1X Asp35(H)	1X His34(H)<br>1X lys35(H)	1X His34(H)	1X His34(H)	1X His34(H)	1X His34(H)
Gly496	1X Lys353(P)<br>1X Asp38(H)<br>2X Lys353(H)								
Gln498	1X Gln42(P)<br>3X Tyr41(H)<br>2X Gln42(H)<br>1X Leu45(H)								
Thr500	1X Tyr41(P)<br>3X Tyr41(H)<br>1X Asn330(H)<br>2X Asp355(H)<br>2X Arg357(H)	1X Aal355(H)	1X Asn355(P)<br>4X Aal355(H)						
Asn501	3X Tyr41(H)<br>1X Lys353(H)		1X Aal355(H)						
Gly502	1X Lys353(P)<br>1X Lys353(H)<br>2X Gly354(H)								
Tyr505	5X Lys353(H)<br>1X Gly354(H)								

Supplementary Table 2: (Continued)

Spike RBD Wuhan strain contact residues	hACE2 isoform 1 contact residues	L455F							
		D355A	D355N	E35D	E35K	F40L	M82I	T27A	S19P
Lys417	1X Asp30(P)<br>1X Asp30(H)								
Gly446	1X Gln42(P)								
Tyr449	1X Asp38(P)<br>1X Gln42(P)<br>3X Asp38(H)								
Tyr453	1X His34(P)<br>2X His34(H)								
Leu455	4X His34(H)	4X Asp30(H)<br>1X Lys 31(H)	4X Asp30(H)<br>1X Lys 31(H)	4X Asp30(H)<br>1X Lys 31(H)	4X Asp30(H)<br>1X Lys 31(H)	4X Asp30(H)<br>1X Lys 31(H)	4X Asp30(H)<br>1X Lys 31(H)	4X Asp30(H)<br>1X Lys 31(H)	4X Asp30(H)<br>1X Lys 31(H)
Phe456	1X Thr27(H)<br>1X Asp30(H)							1X Ala27(H)	
Ala475	1X Ser19(P)<br>2X Ser19(H)<br>1X Gln24(H)								Missing<br>1X Pro19(H)
Gly476	1X Ser19(H)								1X Pro19(H)
Phe486	1X Met82(H)<br>4X Tyr83(H)						1X Ile82(H)		
Asn487	1X Gln24(P)<br>1X Tyr83(P)<br>6X Gln24(H)<br>3X Tyr83(H)								
Tyr489	1X Thr27(H)<br>1X Phe28(H)							Missing	
Gln493	2X His34(H)<br>1X Glu35(H)			1X Asp35(P)<br>1X Asp35(H)	1X Lys35(H)				
Gly496	1X Lys353(P)<br>1X Asp38(H)<br>2X Lys353(H)								
Gln498	1X Gln42(P)<br>3X Tyr41(H)<br>2X Gln42(H)<br>1X Leu45(H)								
Thr500	1X Tyr41(P)<br>3X Tyr41(H)<br>1X Asn330(H)<br>2X Asp355(H)<br>2X Arg357(H)	1X Ala355(H)	1X Asn335(P)<br>4X Asn355(H)						
Asn501	3X Tyr41(H)<br>1X Lys353(H)		1X Asn355(H)						
Gly502	1X Lys353(P)<br>1X Lys353(H)<br>2X Gly354(H)								
Tyr505	5X Lys353(H)<br>1X Gly354(H)								

Supplementary Table 2: (Continued)

Spike RBD Wuhan strain contact residues	hACE2 isoform 1 contact residues	F456L							
		D355A	D355N	E35D	E35K	F40L	M82I	T27A	S19P
Lys417	1X Asp30(P)<br>1X Asp30(H)								
Gly446	1X Gln42(P)								
Tyr449	1X Asp38(P)<br>1X Gln42(P)<br>3X Asp38(H)								
Tyr453	1X His34(P)<br>2X His34(H)								
Leu455	4X His34(H)								
Phe456	1X Thr27(H)<br>1X Asp30(H)	Missing	Missing	Missing	Missing	Missing	Missing	Missing<br>Missing	Missing
Ala475	1X Ser19(P)<br>2X Ser19(H)<br>1X Gln24(H)								Missing<br>1X Pro19(H)
Gly476	1X Ser19(H)								1X Pro19(H)
Phe486	1X Met82(H)<br>4X Tyr83(H)						1X Ile82(H)		
Asn487	1X Gln24(P)<br>1X Tyr83(P)<br>6X Gln24(H)<br>3X Tyr83(H)								
Tyr489	1X Thr27(H)<br>1X Phe28(H)							Missing	
Gln493	2X His34(H)<br>1X Glu35(H)			1X Asp35(P)<br>1X Asp35(H)	1X Lys35(H)				
Gly496	1X Lys353(P)<br>1X Asp38(H)<br>2X Lys353(H)								
Gln498	1X Gln42(P)<br>3X Tyr41(H)<br>2X Gln42(H)<br>1X Leu45(H)								
Thr500	1X Tyr41(P)<br>3X Tyr41(H)<br>1X Asn330(H)<br>2X Asp355(H)<br>2X Arg357(H)	1X Ala355(H)	1X Asn355(P)<br>4X Asn355(H)						
Asn501	3X Tyr41(H)<br>1X Lys353(H)		1X Ala355(H)						
Gly502	1X Lys353(P)<br>1X Lys353(H)<br>2X Gly354(H)								
Tyr505	5X Lys353(H)<br>1X Gly354(H)								

Supplementary Table 2: (Continued)

Spike RBD Wuhan strain contact residues	hACE2 isoform 1 contact residues	A475V							
		D355A	D355N	E35D	E35K	F40L	M82I	T27A	S19P
Lys417	1X Asp30(P)<br>1X Asp30(H)								
Gly446	1X Gln42(P)								
Tyr449	1X Asp38(P)<br>1X Gln42(P)<br>3X Asp38(H)								
Tyr453	1X His34(P)<br>2X His34(H)								
Leu455	4X His34(H)								
Phe456	1X Thr27(H)<br>1X Asp30(H)								
Ala475	1X Ser19(P)<br>2X Ser19(H)<br>1X Gln24(H)	2X Gln24(H)<br>2X Thr27(H)	2X Gln24(H)<br>2X Thr27(H)	2X Gln24(H)<br>2X Thr27(H)	2X Gln24(H)<br>2X Thr27(H)	2X Gln24(H)<br>2X Thr27(H)	2X Gln24(H)<br>2X Thr27(H)	2X Gln24(H)<br>1X Thr27(H)	1X Pro19(H)<br>2X Gln24(H)<br>1X Thr27(H)
Gly476	1X Ser19(H)								1X Pro19(H)
Phe486	1X Met82(H)<br>4X Tyr83(H)						1X Ile82(H)		
Asn487	1X Gln24(P)<br>1X Tyr83(P)<br>6X Gln24(H)<br>3X Tyr83(H)								
Tyr489	1X Thr27(H)<br>1X Phe28(H)								
Gln493	2X His34(H)<br>1X Glu35(H)			1X Asp35(P)<br>1X Asp35(H)	1X Lys35(H)			Missing	
Gly496	1X Lys353(P)<br>1X Asp38(H)<br>2X Lys353(H)								
Gln498	1X Gln42(P)<br>3X Tyr41(H)<br>2X Gln42(H)<br>1X Leu45(H)								
Thr500	1X Tyr41(P)<br>3X Tyr41(H)<br>1X Asn330(H)<br>2X Asp355(H)<br>2X Arg357(H)	1X Ala355(H)	1X Asn355(P)<br>4X Asn355(H)						
Asn501	3X Tyr41(H)<br>1X Lys353(H)		1X Asn355(H)						
Gly502	1X Lys353(P)<br>1X Lys353(H)<br>2X Gly354(H)								
Tyr505	5X Lys353(H)<br>1X Gly354(H)								

Supplementary Table 2: (Continued)

Spike RBD Wuhan strain contact residues	hACE2 isoform 1 contact residues	G476S							
		D355A	D355N	E35D	E35K	F40L	M82I	T27A	S19P
Lys417	1X Asp30(P)<br>1X Asp30(H)								
Gly446	1X Gln42(P)								
Tyr449	1X Asp38(P)<br>1X Gln42(P)<br>3X Asp38(H)								
Tyr453	1X His34(P)<br>2X His34(H)								
Leu455	4X His34(H)								
Phe456	1X Thr27(H)<br>1X Asp30(H)							1X Ala27(H)	
Ala475	1X Ser19(P)<br>2X Ser19(H)<br>1X Gln24(H)								Missing<br>1X Pro19(H)
Gly476	1X Ser19(H)	Missing	Missing	Missing	Missing	Missing	Missing	Missing	Missing
Phe486	1X Met82(H)<br>4X Tyr83(H)						1X Ile82(H)		
Asn487	1X Gln24(P)<br>1X Tyr83(P)<br>6X Gln24(H)<br>3X Tyr83(H)								
Tyr489	1X Thr27(H)<br>1X Phe28(H)							Missing	
Gln493	2X His34(H)<br>1X Glu35(H)			1X Asp35(P)<br>1X Asp35(H)	1X Lys35(H)				
Gly496	1X Lys353(P)<br>1X Asp38(H)<br>2X Lys353(H)								
Gln498	1X Gln42(P)<br>3X Tyr41(H)<br>2X Gln42(H)<br>1X Leu45(H)								
Thr500	1X Tyr41(P)<br>3X Tyr41(H)<br>1X Asn330(H)<br>2X Asp355(H)<br>2X Arg357(H)	1X Ala355(H)	1X Asn335(P)<br>4X Asn355(H)						
Asn501	3X Tyr41(H)<br>1X Lys353(H)		1X Asn355(H)						
Gly502	1X Lys353(P)<br>1X Lys353(H)<br>2X Gly354(H)								
Tyr505	5X Lys353(H)<br>1X Gly354(H)								

Supplementary Table 2: (Continued)

Spike RBD Wuhan strain contact residues	hACE2 isoform 1 contact residues	G476A							
		D355A	D355N	E35D	E35K	F40L	M82I	T27A	S19P
Lys417	1X Asp30(P)<br>1X Asp30(H)								
Gly446	1X Gln42(P)								
Tyr449	1X Asp38(P)<br>1X Gln42(P)<br>3X Asp38(H)								
Tyr453	1X His34(P)<br>2X His34(H)								
Leu455	4X His34(H)								
Phe456	1X Thr27(H)<br>1X Asp30(H)							1X Ala27(H)	
Ala475	1X Ser19(P)<br>2X Ser19(H)<br>1X Gln24(H)								Missing<br>1X Pro19(H)
Gly476	1X Ser19(H)	Missing	Missing	Missing	Missing	Missing	Missing	Missing	Missing
Phe486	1X Met82(H)<br>4X Tyr83(H)						1X Ile82(H)		
Asn487	1X Gln24(P)<br>1X Tyr83(P)<br>6X Gln24(H)<br>3X Tyr83(H)								
Tyr489	1X Thr27(H)<br>1X Phe28(H)							Missing	
Gln493	2X His34(H)<br>1X Glu35(H)			1X Asp35(P)<br>1X Asp35(H)	1X Lys35(H)				
Gly496	1X Lys353(P)<br>1X Asp38(H)<br>2X Lys353(H)								
Gln498	1X Gln42(P)<br>3X Tyr41(H)<br>2X Gln42(H)<br>1X Leu45(H)								
Thr500	1X Tyr41(P)<br>3X Tyr41(H)<br>1X Asn330(H)<br>2X Asp355(H)<br>2X Arg357(H)	1X Ala355(H)	1X Asn335(P)<br>4X Asn355(H)						
Asn501	3X Tyr41(H)<br>1X Lys353(H)		1X Asn355(H)						
Gly502	1X Lys353(P)<br>1X Lys353(H)<br>2X Gly354(H)								
Tyr505	5X Lys353(H)<br>1X Gly354(H)								

Supplementary Table 2: (Continued)

Spike RBD Wuhan strain contact residues	hACE2 isoform 1 contact residues	F486L							
		D355A	D355N	E35D	E35K	F40L	M82I	T27A	S19P
Lys417	1X Asp30(P)<br>1X Asp30(H)								
Gly446	1X Gln42(P)								
Tyr449	1X Asp38(P)<br>1X Gln42(P)<br>3X Asp38(H)								
Tyr453	1X His34(P)<br>2X His34(H)								
Leu455	4X His34(H)								
Phe456	1X Thr27(H)<br>1X Asp30(H)							1X Ala27(H)	
Ala475	1X Ser19(P)<br>2X Ser19(H)<br>1X Gln24(H)								Missing<br>1X Pro19(H)
Gly476	1X Ser19(H)								1X Pro19(H)
Phe486	1X Met82(H)<br>4X Tyr83(H)	1X Tyr83(H)	1X Tyr83(H)	1X Tyr83(H)	1X Tyr83(H)	1X Tyr83(H)	1X Ile82(H)<br>1X Tyr83(H)	1X Tyr83(H)	1X Tyr83(H)
Asn487	1X Gln24(P)<br>1X Tyr83(P)<br>6X Gln24(H)<br>3X Tyr83(H)								
Tyr489	1X Thr27(H)<br>1X Phe28(H)							Missing	
Gln493	2X His34(H)<br>1X Glu35(H)			1X Asp35(P)<br>1X Asp35(H)	1X Lys35(H)				
Gly496	1X Lys353(P)<br>1X Asp38(H)<br>2X Lys353(H)								
Gln498	1X Gln42(P)<br>3X Tyr41(H)<br>2X Gln42(H)<br>1X Leu45(H)								
Thr500	1X Tyr41(P)<br>3X Tyr41(H)<br>1X Asn330(H)<br>2X Asp355(H)<br>2X Arg357(H)	1X Asp355(H)	1X Asn355(P)<br>4X Asn355(H)						
Asn501	3X Tyr41(H)<br>1X Lys353(H)		1X Asn355(H)						
Gly502	1X Lys353(P)<br>1X Lys353(H)<br>2X Gly354(H)								
Tyr505	5X Lys353(H)<br>1X Gly354(H)								

Supplementary Table 2: (Continued)

Spike RBD Wuhan strain contact residues	hACE2 isoform 1 contact residues	Q493R							
		D355A	D355N	E35D	E35K	F40L	M82I	T27A	S19P
Lys417	1X Asp30(P)<br>1X Asp30(H)								
Gly446	1X Gln42(P)								
Tyr449	1X Asp38(P)<br>1X Gln42(P)<br>3X Asp38(H)								
Tyr453	1X His34(P)<br>2X His34(H)								
Leu455	4X His34(H)								
Phe456	1X Thr27(H)<br>1X Asp30(H)							1X Ala27(H)	
Ala475	1X Ser19(P)<br>2X Ser19(H)<br>1X Gln24(H)								Missing<br>1X Pr019(H)
Gly476	1X Ser19(H)								1X Pro19(H)
Phe486	1X Met82(H)<br>4X Tyr83(H)						1X Ile82(H)		
Asn487	1X Gln24(P)<br>1X Tyr83(P)<br>6X Gln24(H)<br>3X Tyr83(H)								
Tyr489	1X Thr27(H)<br>1X Phe28(H)							Missing	
Gln493	2X His34(H)<br>1X Glu35(H)	1X Glu35 (P)<br>Missing<br>4X Glu35(H)	1X Glu35 (P)<br>Missing<br>4X Glu35(H)	Missing<br>Missing<br>***1X Asp35 (*)***	Missing<br>Missing	1X Glu35 (P)<br>Missing<br>4X Glu35(H)	1X Glu35 (P)<br>Missing<br>4X Glu35(H)	1X Glu35 (P)<br>Missing<br>4X Glu35(H)	1X Glu35 (P)<br>Missing<br>4X Glu35(H)
Gly496	1X Lys353(P)<br>1X Asp38(H)<br>2X Lys353(H)								
Gln498	1X Gln42(P)<br>3X Tyr41(H)<br>2X Gln42(H)<br>1X Leu45(H)								
Thr500	1X Tyr41(P)<br>3X Tyr41(H)<br>1X Asn330(H)<br>2X Asp355(H)<br>2X Arg357(H)	1X Asp355(H)	1X Asn355(P)<br>4X Asn355(H)						
Asn501	3X Tyr41(H)<br>1X Lys353(H)		1X Asn355(H)						
Gly502	1X Lys353(P)<br>1X Lys353(H)<br>2X Gly354(H)								
Tyr505	5X Lys353(H)<br>1X Gly354(H)								

Supplementary Table 2: (Continued)

Spike RBD Wuhan strain contact residues	hACE2 isoform 1 contact residues	Q493L							
		D355A	D355N	E35D	E35K	F40L	M82I	T27A	S19P
Lys417	1X Asp30(P)<br>1X Asp30(H)								
Gly446	1X Gln42(P)								
Tyr449	1X Asp38(P)<br>1X Gln42(P)<br>3X Asp38(H)								
Tyr453	1X His34(P)<br>2X His34(H)								
Leu455	4X His34(H)								
Phe456	1X Thr27(H)<br>1X Asp30(H)							1X Ala27(H)	
Ala475	1X Ser19(P)<br>2X Ser19(H)<br>1X Gln24(H)								Missing<br>1X Pr019(H)
Gly476	1X Ser19(H)								1X Pro19(H)
Phe486	1X Met82(H)<br>4X Tyr83(H)						1X Ile82(H)		
Asn487	1X Gln24(P)<br>1X Tyr83(P)<br>6X Gln24(H)<br>3X Tyr83(H)								
Tyr489	1X Thr27(H)<br>1X Phe28(H)							Missing	
Gln493	2X His34(H)<br>1X Glu35(H)	1X His34(H)<br>Missing<br>1X Lys31(H)	1X His34(H)<br>Missing<br>1X Lys31(H)	1X His34(H)<br>Missing<br>1X lys31(H)	1X His34(H)<br>Missing<br>1X lys31(H)	1X His34(H)<br>Missing<br>1X Lys31(H)	1X His34(H)<br>Missing<br>1X Lys31(H)	1X His34(H)<br>Missing<br>1X Lys31(H)	1X His34(H)<br>Missing<br>1X Lys31(H)
Gly496	1X Lys353(P)<br>1X Asp38(H)<br>2X Lys353(H)								
Gln498	1X Gln42(P)<br>3X Tyr41(H)<br>2X Gln42(H)<br>1X Leu45(H)								
Thr500	1X Tyr41(P)<br>3X Tyr41(H)<br>1X Asn330(H)<br>2X Asp355(H)<br>2X Arg357(H)	1X Asp355(H)	1X Asn355(P)<br>4X Asn355(H)						
Asn501	3X Tyr41(H)<br>1X Lys353(H)		1X Asn355(H)						
Gly502	1X Lys353(P)<br>1X Lys353(H)<br>2X Gly354(H)								
Tyr505	5X Lys353(H)<br>1X Gly354(H)								

Supplementary Table 2: (Continued)

Spike RBD Wuhan strain contact residues	hACE2 isoform 1 contact residues	G496C							
		**D355A**	**D355N**	**E35D**	**E35K**	**F40L**	**M82I**	**T27A**	**S19P**
Lys417	1X Asp30(P)<br>1X Asp30(H)								
Gly446	1X Gln42(P)								
Tyr449	1X Asp38(P)<br>1X Gln42(P)<br>3X Asp38(H)								
Tyr453	1X His34(P)<br>2X His34(H)								
Leu455	4X His34(H)								
Phe456	1X Thr27(H)<br>1X Asp30(H)							1X Ala27(H)	
Ala475	1X Ser19(P)<br>2X Ser19(H)<br>1X Gln24(H)								Missing<br>1X Pr019(H)
Gly476	1X Ser19(H)								1X Pro19(H)
Phe486	1X Met82(H)<br>4X Tyr83(H)						1X Ile82(H)		
Asn487	1X Gln24(P)<br>1X Tyr83(P)<br>6X Gln24(H)<br>3X Tyr83(H)								
Tyr489	1X Thr27(H)<br>1X Phe28(H)							Missing	
Gln493	2X His34(H)<br>1X Glu35(H)			1XAsp35(P)<br>1X Asp35(H)	1X Lys35(H)				
Gly496	1X Lys353(P)<br>1X Asp38(H)<br>2X Lys353(H)	7X Asp38(H)	7X Asn38(H)	7X Asp38(H)	7X Asp38(H)	7X Asp38(H)	7X Asp38(H)	7X Asp38(H)	7X Asp38(H)
Gln498	1X Gln42(P)<br>3X Tyr41(H)<br>2X Gln42(H)<br>1X Leu45(H)								
Thr500	1X Tyr41(P)<br>3X Tyr41(H)<br>1X Asn330(H)<br>2X Asp355(H)<br>2X Arg357(H)	1X Asp355(H)	1X Asn355(P)<br>4X Asn355(H)						
Asn501	3X Tyr41(H)<br>1X Lys353(H)		1X Asn355(H)						
Gly502	1X Lys353(P)<br>1X Lys353(H)<br>2X Gly354(H)								
Tyr505	5X Lys353(H)<br>1X Gly354(H)								

Supplementary Table 2: (Continued)

Spike RBD Wuhan strain contact residues	hACE2 isoform 1 contact residues	T500I							
		D355A	D355N	E35D	E35K	F40L	M82I	T27A	S19P
Lys417	1X Asp30(P)<br>1X Asp30(H)								
Gly446	1X Gln42(P)								
Tyr449	1X Asp38(P)<br>1X Gln42(P)<br>3X Asp38(H)								
Tyr453	1X His34(P)<br>2X His34(H)								
Leu455	4X His34(H)								
Phe456	1X Thr27(H)<br>1X Asp30(H)							1X Ala27(H)	
Ala475	1X Ser19(P)<br>2X Ser19(H)<br>1X Gln24(H)								Missing<br>1X Pr019(H)
Gly476	1X Ser19(H)								1X Pro19(H)
Phe486	1X Met82(H)<br>4X Tyr83(H)						1X Ile82(H)		
Asn487	1X Gln24(P)<br>1X Tyr83(P)<br>6X Gln24(H)<br>3X Tyr83(H)								
Tyr489	1X Thr27(H)<br>1X Phe28(H)							Missing	
Gln493	2X His34(H)<br>1X Glu35(H)			1XAsp35(P)<br>1X Asp35(H)	1X Lys35(H)				
Gly496	1X Lys353(P)<br>1X Asp38(H)<br>2X Lys353(H)								
Gln498	1X Gln42(P)<br>3X Tyr41(H)<br>2X Gln42(H)<br>1X Leu45(H)	2X Tyr41(H)	2X Tyr41(H)	2X Tyr41(H)	2X Tyr41(H)	2X Tyr41(H)	2X Tyr41(H)	2X Tyr41(H)	2X Tyr41(H)
Thr500	1X Tyr41(P)<br>3X Tyr41(H)<br>1X Asn330(H)<br>2X Asp355(H)<br>2X Arg357(H)	Missing<br>1X Tyr41(H)<br>Missing<br>1X Arg357(H)<br>1X Leu45(H)	Missing<br>1X Tyr41(H)<br>1X Asp355(H)<br>1X Arg357(H)<br>1X Leu45(H)	Missing<br>1X Tyr41(H)<br>1X Asp355(H)<br>1X Arg357(H)<br>1X Leu45(H)	Missing<br>1X Tyr41(H)<br>1X Asp355(H)<br>1X Arg357(H)<br>1X Leu45(H)	Missing<br>1X Tyr41(H)<br>1X Asp355(H)<br>1X Arg357(H)<br>1X Leu45(H)	Missing<br>1X Tyr41(H)<br>1X Asp355(H)<br>1X Arg357(H)<br>1X Leu45(H)	Missing<br>1X Tyr41(H)<br>1X Asp355(H)<br>1X Arg357(H)<br>1X Leu45(H)	Missing<br>1X Tyr41(H)<br>1X Asp355(H)<br>1X Arg357(H)<br>1X Leu45(H)
Asn501	3X Tyr41(H)<br>1X Lys353(H)	1X Tyr41(H)	1X Tyr41(H)<br>1X Asn355(H)	1X Tyr41(H)	1X Tyr41(H)	1X Tyr41(H)	1X Tyr41(H)	1X Tyr41(H)	1X Tyr41(H)
Gly502	1X Lys353(P)<br>1X Lys353(H)<br>2X Gly354(H)								
Tyr505	5X Lys353(H)<br>1X Gly354(H)								

Supplementary Table 2: (Continued)

Spike RBD Wuhan strain contact residues	hACE2 isoform 1 contact residues	N501T							
		D355A	D355N	E35D	E35K	F40L	M82I	T27A	S19P
Lys417	1X Asp30(P)<br>1X Asp30(H)								
Gly446	1X Gln42(P)								
Tyr449	1X Asp38(P)<br>1X Gln42(P)<br>3X Asp38(H)								
Tyr453	1X His34(P)<br>2X His34(H)								
Leu455	4X His34(H)								
Phe456	1X Thr27(H)<br>1X Asp30(H)							1X Ala27(H)	
Ala475	1X Ser19(P)<br>2X Ser19(H)<br>1X Gln24(H)								Missing<br>1X Pr019(H)
Gly476	1X Ser19(H)								1X Pro19(H)
Phe486	1X Met82(H)<br>4X Tyr83(H)						1X Ile82(H)		
Asn487	1X Gln24(P)<br>1X Tyr83(P)<br>6X Gln24(H)<br>3X Tyr83(H)								
Tyr489	1X Thr27(H)<br>1X Phe28(H)							Missing	
Gln493	2X His34(H)<br>1X Glu35(H)			1XAsp35(P)<br>1X Asp35(H)	1X Lys35(H)				
Gly496	1X Lys353(P)<br>1X Asp38(H)<br>2X Lys353(H)								
Gln498	1X Gln42(P)<br>3X Tyr41(H)<br>2X Gln42(H)<br>1X Leu45(H)								
Thr500	1X Tyr41(P)<br>3X Tyr41(H)<br>1X Asn330(H)<br>2X Asp355(H)<br>2X Arg357(H)	1X Ala355(H)	1X Asn355(P)<br>4X Asn355(H)						
Asn501	3X Tyr41(H)<br>1X Lys353(H)	2X Tyr41(H)<br>3X Lys353(H)	2X Tyr41(H)<br>3X Lys353(H)<br>1XAsn355(H)	2X Tyr41(H)<br>3X Lys353(H)	2X Tyr41(H)<br>3X Lys353(H)	2X Tyr41(H)<br>3X Lys353(H)	2X Tyr41(H)<br>3X Lys353(H)	2X Tyr41(H)<br>3X Lys353(H)	2X Tyr41(H)<br>3X Lys353(H)
Gly502	1X Lys353(P)<br>1X Lys353(H)<br>2X Gly354(H)								
Tyr505	5X Lys353(H)<br>1X Gly354(H)								

Supplementary Table 2: (Continued)

Spike RBD Wuhan strain contact residues	hACE2 isoform 1 contact residues	N501S							
		D355A	D355N	E35D	E35K	F40L	M82I	T27A	S19P
Lys417	1X Asp30(P)<br>1X Asp30(H)								
Gly446	1X Gln42(P)								
Tyr449	1X Asp38(P)<br>1X Gln42(P)<br>3X Asp38(H)								
Tyr453	1X His34(P)<br>2X His34(H)								
Leu455	4X His34(H)								
Phe456	1X Thr27(H)<br>1X Asp30(H)							1X Ala27(H)	
Ala475	1X Ser19(P)<br>2X Ser19(H)<br>1X Gln24(H)								Missing<br>1X Pr019(H)
Gly476	1X Ser19(H)								1X Pro19(H)
Phe486	1X Met82(H)<br>4X Tyr83(H)						1X Ile82(H)		
Asn487	1X Gln24(P)<br>1X Tyr83(P)<br>6X Gln24(H)<br>3X Tyr83(H)								
Tyr489	1X Thr27(H)<br>1X Phe28(H)							Missing	
Gln493	2X His34(H)<br>1X Glu35(H)			1XAsp35(P)<br>1X Asp35(H)	1X Lys35(H)				
Gly496	1X Lys353(P)<br>1X Asp38(H)<br>2X Lys353(H)								
Gln498	1X Gln42(P)<br>3X Tyr41(H)<br>2X Gln42(H)<br>1X Leu45(H)								
Thr500	1X Tyr41(P)<br>3X Tyr41(H)<br>1X Asn330(H)<br>2X Asp355(H)<br>2X Arg357(H)	1X Ala355(H)	1X Asn355(P)<br>4X Asn355(H)						
Asn501	3X Tyr41(H)<br>1X Lys353(H)	1X Tyr41(H)<br>3X Lys353(H)	1X Tyr41(H)<br>3X Lys353(H)<br>1XAsn355(H)	1X Tyr41(H)<br>3X Lys353(H)	1X Tyr41(H)<br>3X Lys353(H)	1X Tyr41(H)<br>3X Lys353(H)	1X Tyr41(H)<br>3X Lys353(H)	1X Tyr41(H)<br>3X Lys353(H)	1X Tyr41(H)<br>3X Lys353(H)
Gly502	1X Lys353(P)<br>1X Lys353(H)<br>2X Gly354(H)								
Tyr505	5X Lys353(H)<br>1X Gly354(H)								

Supplementary Table 2: (Continued)

Spike RBD Wuhan strain contact residues	hACE2 isoform 1 contact residues	N501Y							
		D355A	D355N	E35D	E35K	F40L	M82I	T27A	S19P
Lys417	1X Asp30(P)<br>1X Asp30(H)								
Gly446	1X Gln42(P)								
Tyr449	1X Asp38(P)<br>1X Gln42(P)<br>3X Asp38(H)								
Tyr453	1X His34(P)<br>2X His34(H)								
Leu455	4X His34(H)								
Phe456	1X Thr27(H)<br>1X Asp30(H)							1X Ala27(H)	
Ala475	1X Ser19(P)<br>2X Ser19(H)<br>1X Gln24(H)								Missing<br>1X Pr019(H)
Gly476	1X Ser19(H)								1X Pro19(H)
Phe486	1X Met82(H)<br>4X Tyr83(H)						1X Ile82(H)		
Asn487	1X Gln24(P)<br>1X Tyr83(P)<br>6X Gln24(H)<br>3X Tyr83(H)								
Tyr489	1X Thr27(H)<br>1X Phe28(H)							Missing	
Gln493	2X His34(H)<br>1X Glu35(H)			1XAsp35(P)<br>1X Asp35(H)	1X Lys35(H)				
Gly496	1X Lys353(P)<br>1X Asp38(H)<br>2X Lys353(H)								
Gln498	1X Gln42(P)<br>3X Tyr41(H)<br>2X Gln42(H)<br>1X Leu45(H)								
Thr500	1X Tyr41(P)<br>3X Tyr41(H)<br>1X Asn330(H)<br>2X Asp355(H)<br>2X Arg357(H)	1X Ala355(H)	1X Asn355(P)<br>4X Asn355(H)						
Asn501	3X Tyr41(H)<br>1X Lys353(H)	5X Tyr41(H)<br>6X Lys353(H)	5X Tyr41(H)<br>6X Lys353(H)<br>1XAsn355(H)	5X Tyr41(H)<br>6X Lys353(H)	5X Tyr41(H)<br>6X Lys353(H)	5X Tyr41(H)<br>6X Lys353(H)	5X Tyr41(H)<br>6X Lys353(H)	5X Tyr41(H)<br>6X Lys353(H)	5X Tyr41(H)<br>6X Lys353(H)
Gly502	1X Lys353(P)<br>1X Lys353(H)<br>2X Gly354(H)								
Tyr505	5X Lys353(H)<br>1X Gly354(H)								

Supplementary Table 2: (Continued)

Spike RBD Wuhan strain contact residues	hACE2 isoform 1 contact residues	G502D							
		**D355A**	**D355N**	**E35D**	**E35K**	**F40L**	**M82I**	**T27A**	**S19P**
Lys417	1X Asp30(P)<br>1X Asp30(H)								
Gly446	1X Gln42(P)								
Tyr449	1X Asp38(P)<br>1X Gln42(P)<br>3X Asp38(H)								
Tyr453	1X His34(P)<br>2X His34(H)								
Leu455	4X His34(H)								
Phe456	1X Thr27(H)<br>1X Asp30(H)							1X Ala27(H)	
Ala475	1X Ser19(P)<br>2X Ser19(H)<br>1X Gln24(H)								Missing<br>1X Pr019(H)
Gly476	1X Ser19(H)								1X Pro19(H)
Phe486	1X Met82(H)<br>4X Tyr83(H)						1X Ile82(H)		
Asn487	1X Gln24(P)<br>1X Tyr83(P)<br>6X Gln24(H)<br>3X Tyr83(H)								
Tyr489	1X Thr27(H)<br>1X Phe28(H)							Missing	
Gln493	2X His34(H)<br>1X Glu35(H)			1XAsp35(P)<br>1X Asp35(H)	1X Lys35(H)				
Gly496	1X Lys353(P)<br>1X Asp38(H)<br>2X Lys353(H)								
Gln498	1X Gln42(P)<br>3X Tyr41(H)<br>2X Gln42(H)<br>1X Leu45(H)								
Thr500	1X Tyr41(P)<br>3X Tyr41(H)<br>1X Asn330(H)<br>2X Asp355(H)<br>2X Arg357(H)	1X Ala355(H)	1X Asn355(P)<br>4X Asn355(H)						
Asn501	3X Tyr41(H)<br>1X Lys353(H)		1XAsn355(H)						
Gly502	1X Lys353(P)<br>1X Lys353(H)<br>2X Gly354(H)	2X Lys353(H)<br>4X Gly354(H)<br>1X Thr324(H)	2X Lys353(H)<br>4X Gly354(H)<br>1X Thr324(H)	2X Lys353(H)<br>4X Gly354(H)<br>1X Thr324(H)	2X Lys353(H)<br>4X Gly354(H)<br>1X Thr324(H)	2X Lys353(H)<br>4X Gly354(H)<br>1X Thr324(H)	2X Lys353(H)<br>4X Gly354(H)<br>1X Thr324(H)	2X Lys353(H)<br>4X Gly354(H)<br>1X Thr324(H)	2X Lys353(H)<br>4X Gly354(H)<br>1X Thr324(H)
Tyr505	5X Lys353(H)<br>1X Gly354(H)								

Supplementary Table 2: (Continued)

Spike RBD Wuhan strain contact residues	hACE2 isoform 1 contact residues	G502C							
		D355A	D355N	E35D	E35K	F40L	M82I	T27A	S19P
Lys417	1X Asp30(P)<br>1X Asp30(H)								
Gly446	1X Gln42(P)								
Tyr449	1X Asp38(P)<br>1X Gln42(P)<br>3X Asp38(H)								
Tyr453	1X His34(P)<br>2X His34(H)								
Leu455	4X His34(H)								
Phe456	1X Thr27(H)<br>1X Asp30(H)							1X Ala27(H)	
Ala475	1X Ser19(P)<br>2X Ser19(H)<br>1X Gln24(H)								Missing<br>1X Pr019(H)
Gly476	1X Ser19(H)								1X Pro19(H)
Phe486	1X Met82(H)<br>4X Tyr83(H)						1X Ile82(H)		
Asn487	1X Gln24(P)<br>1X Tyr83(P)<br>6X Gln24(H)<br>3X Tyr83(H)								
Tyr489	1X Thr27(H)<br>1X Phe28(H)							Missing	
Gln493	2X His34(H)<br>1X Glu35(H)			1XAsp35(P)<br>1X Asp35(H)	1X Lys35(H)				
Gly496	1X Lys353(P)<br>1X Asp38(H)<br>2X Lys353(H)								
Gln498	1X Gln42(P)<br>3X Tyr41(H)<br>2X Gln42(H)<br>1X Leu45(H)								
Thr500	1X Tyr41(P)<br>3X Tyr41(H)<br>1X Asn330(H)<br>2X Asp355(H)<br>2X Arg357(H)	1X Ala355(H)	1X Asn355(P)<br>4X Asn355(H)						
Asn501	3X Tyr41(H)<br>1X Lys353(H)		1XAsn355(H)						
Gly502	1X Lys353(P)<br>1X Lys353(H)<br>2X Gly354(H)	2X Lys353(H)<br>4X Gly354(H)	2X Lys353(H)<br>4X Gly354(H)	2X Lys353(H)<br>4X Gly354(H)	2X Lys353(H)<br>4X Gly354(H)	2X Lys353(H)<br>4X Gly354(H)	2X Lys353(H)<br>4X Gly354(H)	2X Lys353(H)<br>4X Gly354(H)	2X Lys353(H)<br>4X Gly354(H)
Tyr505	5X Lys353(H)<br>1X Gly354(H)								

Supplementary Table 2: (Continued)

Spike RBD Wuhan strain contact residues	hACE2 isoform 1 contact residues	G502R							
		D355A	D355N	E35D	E35K	F40L	M82I	T27A	S19P
Lys417	1X Asp30(P)<br>1X Asp30(H)								
Gly446	1X Gln42(P)								
Tyr449	1X Asp38(P)<br>1X Gln42(P)<br>3X Asp38(H)								
Tyr453	1X His34(P)<br>2X His34(H)								
Leu455	4X His34(H)								
Phe456	1X Thr27(H)<br>1X Asp30(H)							1X Ala27(H)	
Ala475	1X Ser19(P)<br>2X Ser19(H)<br>1X Gln24(H)								Missing<br>1X Pr019(H)
Gly476	1X Ser19(H)								1X Pro19(H)
Phe486	1X Met82(H)<br>4X Tyr83(H)						1X Ile82(H)		
Asn487	1X Gln24(P)<br>1X Tyr83(P)<br>6X Gln24(H)<br>3X Tyr83(H)								
Tyr489	1X Thr27(H)<br>1X Phe28(H)							Missing	
Gln493	2X His34(H)<br>1X Glu35(H)			1XAsp35(P)<br>1X Asp35(H)	1X Lys35(H)				
Gly496	1X Lys353(P)<br>1X Asp38(H)<br>2X Lys353(H)								
Gln498	1X Gln42(P)<br>3X Tyr41(H)<br>2X Gln42(H)<br>1X Leu45(H)								
Thr500	1X Tyr41(P)<br>3X Tyr41(H)<br>1X Asn330(H)<br>2X Asp355(H)<br>2X Arg357(H)	1X Ala355(H)	1X Asn355(P)<br>4X Asn355(H)						
Asn501	3X Tyr41(H)<br>1X Lys353(H)		1XAsn355(H)						
Gly502	1X Lys353(P)<br>1X Lys353(H)<br>2X Gly354(H)	4X Gly354(H)<br>3X Thr324(H)	4X Gly354(H)<br>3X Thr324(H)	4X Gly354(H)<br>3X Thr324(H)	4X Gly354(H)<br>3X Thr324(H)	4X Gly354(H)<br>3X Thr324(H)	4X Gly354(H)<br>3X Thr324(H)	4X Gly354(H)<br>3X Thr324(H)	4X Gly354(H)<br>3X Thr324(H)
Tyr505	5X Lys353(H)<br>1X Gly354(H)								

Supplementary Table 2: (Continued)

Spike RBD Wuhan strain contact residues	hACE2 isoform 1 contact residues	Y505H							
		D355A	D355N	E35D	E35K	F40L	M82I	T27A	S19P
Lys417	1X Asp30(P)<br>1X Asp30(H)								
Gly446	1X Gln42(P)								
Tyr449	1X Asp38(P)<br>1X Gln42(P)<br>3X Asp38(H)								
Tyr453	1X His34(P)<br>2X His34(H)								
Leu455	4X His34(H)								
Phe456	1X Thr27(H)<br>1X Asp30(H)							1X Ala27(H)	
Ala475	1X Ser19(P)<br>2X Ser19(H)<br>1X Gln24(H)								Missing<br>1X Pr019(H)
Gly476	1X Ser19(H)								1X Pro19(H)
Phe486	1X Met82(H)<br>4X Tyr83(H)						1X Ile82(H)		
Asn487	1X Gln24(P)<br>1X Tyr83(P)<br>6X Gln24(H)<br>3X Tyr83(H)								
Tyr489	1X Thr27(H)<br>1X Phe28(H)							Missing	
Gln493	2X His34(H)<br>1X Glu35(H)			1XAsp35(P)<br>1X Asp35(H)	1X Lys35(H)				
Gly496	1X Lys353(P)<br>1X Asp38(H)<br>2X Lys353(H)								
Gln498	1X Gln42(P)<br>3X Tyr41(H)<br>2X Gln42(H)<br>1X Leu45(H)								
Thr500	1X Tyr41(P)<br>3X Tyr41(H)<br>1X Asn330(H)<br>2X Asp355(H)<br>2X Arg357(H)	1X Asp355(H)	1X Asn355(P)<br>4X Asn355(H)						
Asn501	3X Tyr41(H)<br>1X Lys353(H)		1X Asn355(H)						
Gly502	1X Lys353(P)<br>1X Lys353(H)<br>2X Gly354(H)								
Tyr505	5X Lys353(H)<br>1X Gly354(H)	2X Lys353(H)<br>Missing	2X Lys353(H)<br>Missing	2X Lys353(H)<br>Missing	2X Lys353(H)<br>Missing	2X Lys353(H)<br>Missing	2X Lys353(H)<br>Missing	2X Lys353(H)<br>Missing	2X Lys353(H)<br>Missing

Supplementary Table 2: (Continued)

Spike RBD Wuhan strain contact residues	hACE2 isoform 1 contact residues	Y505E							
		D355A	D355N	E35D	E35K	F40L	M82I	T27A	S19P
Lys417	1X Asp30(P)<br>1X Asp30(H)								
Gly446	1X Gln42(P)								
Tyr449	1X Asp38(P)<br>1X Gln42(P)<br>3X Asp38(H)								
Tyr453	1X His34(P)<br>2X His34(H)								
Leu455	4X His34(H)								
Phe456	1X Thr27(H)<br>1X Asp30(H)							1X Ala27(H)	
Ala475	1X Ser19(P)<br>2X Ser19(H)<br>1X Gln24(H)								Missing<br>1X Pr019(H)
Gly476	1X Ser19(H)								1X Pro19(H)
Phe486	1X Met82(H)<br>4X Tyr83(H)						1X Ile82(H)		
Asn487	1X Gln24(P)<br>1X Tyr83(P)<br>6X Gln24(H)<br>3X Tyr83(H)								
Tyr489	1X Thr27(H)<br>1X Phe28(H)							Missing	
Gln493	2X His34(H)<br>1X Glu35(H)			1XAsp35(P)<br>1X Asp35(H)	1X Lys35(H)				
Gly496	1X Lys353(P)<br>1X Asp38(H)<br>2X Lys353(H)								
Gln498	1X Gln42(P)<br>3X Tyr41(H)<br>2X Gln42(H)<br>1X Leu45(H)								
Thr500	1X Tyr41(P)<br>3X Tyr41(H)<br>1X Asn330(H)<br>2X Asp355(H)<br>2X Arg357(H)	1X Asp355(H)	1X Asn355(P)<br>4X Asn355(H)						
Asn501	3X Tyr41(H)<br>1X Lys353(H)		1X Asn355(H)						
Gly502	1X Lys353(P)<br>1X Lys353(H)<br>2X Gly354(H)								
Tyr505	5X Lys353(H)<br>1X Gly354(H)	7X Lys353(H)<br>Missing<br>**_1X Lys353 (*)_**	7X Lys353(H)<br>Missing<br>**_1X Lys353 (*)_**	7X Lys353(H)<br>Missing<br>**_1X Lys353 (*)_**	7X Lys353(H)<br>Missing<br>**_1X Lys353 (*)_**	7X Lys353(H)<br>Missing<br>**_1X Lys353 (*)_**	7X Lys353(H)<br>Missing<br>**_1X Lys353 (*)_**	7X Lys353(H)<br>Missing<br>**_1X Lys353 (*)_**	7X Lys353(H)<br>Missing<br>**_1X Lys353 (*)_**
