# Supplementary material for: A Computational Approach to Evaluate the Combined Effect of SARS-CoV-2 RBD Mutations and ACE2 Receptor Genetic Variants on Infectivity: The COVID-19 Host-Pathogen Nexus
Source: Front Cell Infect Microbiol. 2021 Aug 9;11:707194. doi: 10.3389/fcimb.2021.707194 (PMC8381355; doi:10.3389/fcimb.2021.707194)
Supplement: Supplementary file 7 [file Table_3.docx]

**Supplementary Table 3:** List of the accession numbers of the hACE2 receptor variants. NA: not available.

| hACE2 variant  (amino acid change) | Accession number |
| --- | --- |
| S19P | rs73635825 |
| T27A | rs781255386 |
| E35D | rs778500138 |
| E35K | rs1348114695 |
| F40L | rs924799658 |
| M28I | rs766996587 |
| W163R | rs777568369 |
| Y252N | rs371464495 |
| D355N | rs961360700 |
| D355A | NA |
| G377E | rs767462182 |
| G405E | rs1418150776 |
